# Clinical Potential of Hyperbaric Pressure-Treated Whey Protein

**DOI:** 10.3390/healthcare3020452

**Published:** 2015-06-19

**Authors:** André F. Piccolomini, Stan Kubow, Larry C. Lands

**Affiliations:** 1 Tactio Health Group, 290 Place d’Youville, Montreal, QC H2Y 2B6, Canada; 2 School of Dietetics and Human Nutrition, McGill University, 21,111 Lakeshore, Ste-Anne-de-Bellevue, QC H9X 3V9, Canada; E-Mail: stan.kubow@mcgill.ca; 3 Montreal Children’s Hospital McGill University Health Centre, Division of Pediatric Respiratory Medicine, Room D380, 2300 Tupper Street, Montreal, QC H3H 1P3, Canada; E-Mail: larry.lands@muhc.mcgill.ca

**Keywords:** whey protein isolates, hyperbaric pressure, antioxidant, immunomodulation, nutraceutical properties

## Abstract

Whey protein (WP) from cow’s milk is a rich source of essential and branched chain amino acids. Whey protein isolates (WPI) has been demonstrated to support muscle accretion, antioxidant activity, and immune modulation. However, whey is not readily digestible due to its tight conformational structure. Treatment of WPI with hyperbaric pressure results in protein unfolding. This enhances protein digestion, and results in an altered spectrum of released peptides, and greater release of essential and branched chain amino acids. Pressurized whey protein isolates (pWPI), through a series of cell culture, animal models and clinical studies, have been demonstrated to enhance muscle accretion, reduce inflammation, improve immunity, and decrease fatigue. It is also conceivable that pWPI would be more accessible to digestive enzymes, which would allow for a more rapid proteolysis of the proteins and an increased or altered release of small bioactive peptides. The altered profile of peptides released from WP digestion could thus play a role in the modulation of the immune response and tissue glutathione (GSH) concentrations. The research to date presents potentially interesting applications for the development of new functional foods based on hyperbaric treatment of WPI to produce products with more potent nutritional and nutraceutical properties.

## 1. Introduction

Whey proteins (or milk serum proteins) comprise approximately 20% of total bovine milk proteins [1]. Whey proteins are defined as proteins in milk that remain soluble after acid or rennet casein precipitation [2]. Acid precipitation yields acid whey, while rennet casein precipitation yields sweet or rennet whey [3]. Whey proteins are considered globular proteins whereas their solubility varies over a wide range between pH 2 and 10 [4].

The amino acid composition is the most important factor in defining food protein quality, followed by the digestibility of the protein and the bioavailability of its amino acids. Due to their amino acid composition, the major bovine milk proteins, *i.e.*, caseins and whey proteins are regarded as complete protein sources of essential amino acids (EAA). However, whey proteins are considered superior to other types of proteins such as casein due to their better digestibility, absorption and closer amino acid profile to human requirements [5,6,7]. In addition to the study of their nutritive value, milk proteins are extensively being examined as the primary food sources for a variety of biologically-active peptides used in clinical applications such as hypertension, hypercholesterolemia, cancer, and inflammatory bowel disease (IBD) [8]. The major whey proteins are β-lactoglobulin (β-lb), α-lactalbumin (α-la), serum albumin, immunoglobulins, and glycomacropeptide (GMP), while minor proteins include lactoperoxidase, lactoferrin (Lf), β2-microglobulin, lysozyme, insulin-like growth factor, γ-globulins and several other small proteins [9]. A large number of human and animal feeding trials have indicated that whey proteins exert putative health benefits that include anti-inflammatory, immune-enhancement, antioxidative, and anabolic effects [10].

## 2. The Antioxidant and Anti-Inflammatory Effects of Whey Proteins

Several studies have demonstrated that whey protein intake can exert significant protective effects against oxidative stress imbalances associated with a variety of chronic and acute disease conditions including cancer, heart disease, diabetes, and IBD [11,12]. Differences in EAA composition between whole milk proteins with high biological value can greatly affect their biological and nutritional impact. For example, although both casein and whey are proteins with high biological value, several studies have indicated that the comparatively high concentration of cysteine in whey protein stimulates the synthesis of glutathione (GSH), an intracellular cysteine-containing peptide. This is not true for casein, which does not stimulate GSH [13,14]. The main metabolic fates of cysteine are in synthesis of GSH and specific acute phase proteins such as albumin and haptoglobin [15]. Several studies have shown that sulphur amino acid requirements are increased in stress situations [16,17] and that cysteine supplementation of the diet of septic rats had beneficial effects on recovery of protein status [17]. Other authors suggested that these latter beneficial effects are associated with an increase in GSH synthesis, since GSH turnover may account for more than 50% of cysteine flux in healthy men [18]. Another study has demonstrated that piglets with dextran sodium sulfate (DSS)-induced colitis supplemented with L-cysteine had markedly improved colon histology including lower inflammation, decreased crypt damage, and increased intestinal regeneration [19].

The anti-inflammatory effects of GMP were investigated in a rat model of 2,4,6-trinitrobenzenesulfonic acid to induced colitis. GMP was administered orally to female Wistar rats 3 h post colitis induction. The results showed a decrease in body weight loss, anorexia, colonic damage, colonic alkaline phosphatase activity and IL-1β compared with control rats [20]. Another study investigated the effect of whey protein in a rat model of DSS-induced colitis. Rats were fed diets containing casein (control), whey protein, or casein plus threonine and cysteine (positive control) for 14 day prior to DSS consumption for 7 day. The authors demonstrated that whey protein and positive control diets decreased colonic expression of IL-1β, calprotectin and iNOS, decreased diarrhea, and increased mucin secretion [21]. The anti-oxidative and anti-inflammatory effects of Enprocal, a protein supplement containing a 41% content of whey protein concentrate (WPC), were investigated on gut cell proliferation. Caco-2 cells were treated with digested and undigested Enprocal. The results demonstrated a down regulation of TNF-α and IL-8 and upregulation of IL-2 and IL-10 secretion in Caco-2 cells fed digested Enprocal [22]. These results demonstrated that digested whey protein products exert more potent bioactive properties than whole whey proteins, and promote a more physiological context in the cell culture system. Caco-2 intestinal epithelial cells have been used as an *in vitro* model of the gut epithelium [23]. Caco-2 cell cultures have been used to model IBD. Caco-2 cells exposed to inflammatory mediators such as IL-1β, TNF-α, IFN-γ and lipopolysaccharide (LPS) regulate gut maintenance and has a defence mechanism by controlling the permeability of intestinal epithelial and by acting as inflammatory mediators [24]. The *in vitro* inflammatory response in the intestine is up-regulated by CXCL16 mRNA and protein expression, proposing an important role for this chemokine in the inflammation of the intestine [25].

β-lb is currently thought to be an important source of biologically active peptides that are inactive within the sequence of the precursor protein, but can be released by *in vivo* or *in vitro* enzymatic proteolysis. Once released and absorbed, these peptides may play important roles in human health promoting antihypertensive, antioxidant, and antimicrobial activities [26]. The antioxidant activity of hydrolysates of β-lb was investigated following hydrolysis by different preparations of commercial proteases (pepsin, trypsin, chymotrypsin, thermolysin and corolase). The results demonstrated that a combination of pepsin, trypsin, and chymotrypsin was the most appropriate to produce β-lb hydrolysates having high oxygen radical scavenging activity, measured by oxygen radical absorbance capacity [27]. The whey protein Lf binds to cationic metals such as Fe^+2^, Fe^+3^, Cu^+2^, Zn^+2^, Mn^+2^, and so could play a role in both stable iron delivery and scavenging of free iron and other minerals that would otherwise catalyze oxidative reactions [28]. Moreover, studies carried out in mice have shown that administration of Lf can reduce gastritis and protect gut mucosal integrity during LPS-induced endotoxemia [29,30,31]. The above findings have been documented in a number of cell culture, animal and clinical trials and are summarized in Table 1.

**Table 1:** Antioxidant and anti-inflammatory effects of whey proteins and peptides.

Study Objective	Treatment	Overall Results	Reference
Supplementation with whey protein isolate (WPI) to augment intracellular glutathione (GSH) and enhance performance.	Adults received 20 g of WPI or casein for 3 months.	Enhanced lymphocyte GSH (35%); increased peak power and 30-s work capacity (13% ± 3.7%, *p* < 0.03) in the WPI group.	[32]
Examine the importance of an adequate supply of cysteine and glycine to rats in a low and high protein diets.	Rats on a low protein diet (80 g/kg) supplemented with L-cysteine (4 g/kg) and glycine (5 g/kg), alone or in combination, or a high protein (200 g/kg) diet for 1 week before injection with tumor necrosis factor-alpha (TNF-α) or saline.	Increased liver weight, zinc and GSH concentrations in the high protein-fed rats; greater liver weight after TNF-α treatment in the rats supplemented with glycine and cysteine; enhanced ceruloplasmin, alpha-2-macroglobulin and alpha-1-acid glycoprotein in the TNF-α -treated rats than in saline controls in each dietary group.	[33]
Effect of glycomacropeptide (GMP) in a rat model of trinitrobenzene-sulfonic acid-induced colitis.	GMP orally administered to female Wistar rats 2 day prior or 3 h post colitis induction.	Dose-dependent decrease in body weight loss, anorexia, colonic damage, colonic alkaline phosphatase activity and interleukin-1 beta (IL-1β).	[20]
Effect of dietary cheese whey feeding in a rat model of dextran sodium sulfate (DSS)-induced colitis.	Male Wistar rats fed diets containing casein, cheese whey, or casein + threonine/cysteine for 14 day d prior to DSS consumption for 7 day.	Cheese whey and positive control diets decreased colonic expression of IL-1β, calprotectin and inducible nitric oxide synthase (iNOS); softened stools, decreased diarrhea, and increased mucin secretion.	[21]
Effect of supplementation of L-cysteine in piglets with DSS-induced colitis.	Piglets were fed 0.15 g/kg/day of L-cysteine or saline for 10 d.	Improved colon histology including lower inflammation, decreased crypt damage; increased intestinal regeneration in the piglets-fed L-cysteine.	[19]
Immunomodulatory, antioxidative and anti-inflammatory effects of Enprocal (41% whey protein concentrate (WPC) in gut cell proliferation.	Caco-2 cells treated with digested and undigested Enprocal.	Downregulation of TNF-α and IL-1β, upregulation of IL-2, IL-10 and interferon gamma (IFNγ) secretion; decreased adhesion of Jurkat E6-1 and Tamm-Horsfall Protein (THP)-1 cells to Caco-2 monolayer.	[22]
Effect of bovine and human lactoferrin (Lf) and lactoferricin B on a monocytic cell line.	Lipopolysaccharide (LPS)-stimulated THP-1 cells incubated with Lf or lactoferricin B.	Both Lf and lactoferricin B decreased IL-6 production.	[34]

## 3. Pressurized Whey Protein Isolates: A Novel Concept

High pressure processing is one of the most successful techniques to non-thermally sterilize foods. Although the primary structure of proteins remains intact during pressure treatment [35], high-pressure treatment above 200 MPa can cause changes in the secondary and tertiary structure that can lead to irreversible denaturation of the protein [36]. The pressurized form of whey protein has been investigated as a food ingredient. In the case of milk proteins, high-pressure treatment increases pH, reduces turbidity, changes appearance and can reduce the rennet coagulating time of milk and increase cheese yield, leading to potential applications in the cheese-making industry [37].

An approach used to improve whey protein digestibility is heat treatment. Heat treatment is also used as a sterilization method and it is one of the most important processes involved in the industrial processing of milk and dairy products. The use of temperature at 80 °C for 1 h has been shown to denature and change the conformation of proteins such as β-lb by disrupting the hydrophobic interactions that are the main molecular force holding monomers together. This approach has been shown to facilitate increased *in vitro* and *in vivo* proteolysis of β-lb [38,39]. In the case of infants, however, heat treatment of milk does not seem to result in increased *in vivo* digestion efficiency, possibly due to the fact that the conditions that prevail in the stomach during infancy are not optimal for pepsin activity (pH 3–4) [37,40].

High pressure processing, known also as high hydrostatic or hyperbaric pressure processing, is the process by which a food protein in either liquid or solid state is subjected to pressures of several hundred MPa. Similar to thermal treatment, high pressure treatment can also denature proteins and kill microorganisms with the additional advantages of maintenance of taste and flavour characteristics as this process occurs at low temperatures without any physical damage [41]. In addition to the pressure level, *i.e.*, the degree of pressure applied to a certain food, two modes of pressure can be used by the food industry: static (pressure hold) or dynamic (pressure pulse). The number of pulses used refers to successive series of pressurization and depressurization, and the holding time refers to the length of time a given food is kept under a certain amount of pressure before depressurization [42]. The effects of high pressure on proteins have shown that low-pressure treatment can induce reversible changes such as dissociation of protein-protein complexes, the binding of ligands, and protein conformational changes. In general, protein denaturation occurs when the pressures are higher than about 500 MPa, which is, in most cases, irreversible [42].

It is difficult to establish an overall standard pressurization condition to achieve protein denaturation since the sensitivity of different proteins to high pressure varies and also because different conditions used during high-pressure treatment such as temperature and pH can also change the protein response to the hyperbaric treatment. In the case of whey proteins, β-lb seems to be more sensitive to pressure than bovine serum albumin (BSA) and α-la. It appears that differences in the secondary structure and differences in the number of disulphide bonds in whey proteins play an important role in terms of sensitivity of the protein to pressure treatment. β-lb contains only two disulphide bonds whereas α-la is stabilized by four disulphide bonds and BSA by 17 disulphide bonds [42,43]. Additionally, although β-lb has fewer disulphide bonds than the monomeric protein α-la, it has one -SH (sulfhydryl group) per monomer, which accounts for approximately 90% of the free -SH groups in milk [44]. At neutral pH and dansylated (prepared at atmospheric pressure), α-la structural changes were reversible up to pressures of 400 MPa whereas β-lb lost reversibility of structural changes at 150 MPa or lower. Another study has shown that 400 MPa pressure treatment for 1 h induced the denaturation of β-lb and exposed its single unpaired buried -SH group to the protein surface. The authors verified this finding, by treating the protein with *N*-ethylmaleimide (NEM), an agent which chemically modifies -SH, and analysing the ability of the -SH group to react with 5'dithiobis(2-nitro-benzoic acid) (DTNB). It was concluded that exposure of -SH by pressurization makes the thiol group more reactive and dimerization through intermolecular reaction of -SH occurs [45]. High pressure is one of the most successful techniques that have been used to non-thermally sterilize foods; however, the nutritional implications have received much less attention than those relevant to processing for microbiological safety and preservation. Whether important nutritional implications will arise from new combination uses of physical, natural and conventional food processing techniques is still relatively unexplored [46].

One study has demonstrated that native whey protein isolate was much more resistant to pepsin hydrolysis compared with pressure-treated whey protein isolate. Furthermore, the pressure-treated whey protein isolate showed a more rapid release of absorbable low molecular weight peptides [47]. Since β-lb is the major protein in whey protein isolates, the high resistance of β-lb to pepsin-mediated hydrolysis is likely a major reason for the relatively lesser digestibility of native whey proteins compared with the pressure-treated whey proteins. Numerous studies have demonstrated that there is a low accessibility to peptide bonds localized to the interior of the globular structure of β-lb to hydrolytic action of pepsin [48,49,50,51]. As only the unfolded molecules are susceptible to degradation by proteolytic enzymes [52], it is clear that the increased rate of hydrolysis induced by pressure processing is due to the partial unfolding of β-lb caused by pressure treatment. The unfolding of the protein molecule exposes the hydrophobic amino acids buried in the interior to the proteolytic enzymatic action.

## 4. Discussion

The changes in hyperbaric-induced whey protein structure are associated with the release of unique bioactive peptides as well as improved growth, protein digestibility, and antioxidative effects [47,53]. Representative studies are outlined in Table 2. For the sake of brevity, only the main findings and controversies from animal and human clinical trials are discussed here. The initial studies utilized whey proteins treated by repeated pulse cycling, which provoked changes in protein conformation leading to altered exposure of -SH groups to DTNB [54]. Hyperbaric pressure treatment of WPI was presumed to decrease the surface polarity of the protein molecules thereby exposing the free sulfhydryl groups in the hydrophobic regions to the polar environment.

The effects of a diet of triple cycled whey on the immunoreactivity to whey proteins and antioxidant status of weaned male rats were studied. The rats were fed semi-purified diets containing either pressurized WPI (pWPI) or native WPI at a concentration of 20 wt % for either 17 or 35 day. The pWPI fed animals demonstrated decreased serum IgG and IgE, decreased levels of plasma thiobarbituric acid reactive substances, increased hepatic GSH concentrations, greater weight gain and feed efficiency ratios when compared with the controls fed native WPI [54]. It was suggested that the enhanced weight gain and tissue GSH concentrations were due to an increase in the digestibility of the pWPI; however, effect of pressurization on protein digestibility was not tested.

Electrospray ionization-mass spectrometry studies on whey proteins have shown that holding time and pressure levels could be modified to allow single cycle pressurization to achieve comparable results to multiple cycles of pressurization [55]. In these studies, high hydrostatic pressure treatment of whey proteins resulted in changes in the tertiary structure allowing groups buried in the hydrophobic core of the proteins that were previously inaccessible to the solvent to be charged thereby increasing the charge-state-distribution. Thus, single cycle pressure treatment produced similar partial unfolding of β-lactoglobulin (β-lb), compared to triple cycle pressurization. In addition, the β-lb response to pressure differed between sources of commercial whey. The findings thus indicated that high hydrostatic pressure induced changes in the tertiary structure of whey proteins that were indicative of a relaxation of the native structure of the protein molecules, which could improve the digestibility of the proteins.

An *in vivo* study compared the effect of pWPI using two different modes of treatment: the 3-cycle pressure treatment and 1-cycle pressurization without holding time [56]. Newly weaned male Sprague Dawley rats were fed a diet containing native WPI, 1-cycle or 3-cycle pWPI for 40 day. The 1-cycle pressure treatment group showed significantly greater growth than the group fed native whey protein suggesting that 1-cycle pressure treatment could be used to increase feed efficiency [56]. No differences, however, were observed among the dietary groups in terms of tissue lipid peroxidation indices, liver peroxides, or tissue GSH concentrations, which conflicts with the results from a previous study [54]. These contradictory findings could be a result of the different commercial sources of the native WPI used in the two studies. Many variables associated with commercial WPI production could have led to the differential impact of pressurization in these two animal feeding trials. Functional effects of WPI can be affected by factors such as the choice of isolation processes (membrane filtration or ion exchange) and spray drying techniques used by the manufacturer, whether acid or rennet coagulation was used, as well as batch-to-batch differences [57]. In support of this contention, a murine study noted significant difference in feed efficiency ratio, apparent nitrogen digestibility and muscle GSH content between 1-cycle preparations made from different whey sources [56]. The two commercial sources of WPI have major differences in protein profiles, *i.e.*, β-lb and glycomacropetide content, despite similar sulfur amino acid concentrations, such as the cysteine and methionine content. Further, as noted above, there were differences in β-lb susceptibility to pressure.

The utility of pressurized whey was assessed in two animal studies of lung infection. In mice infected intratracheally with *Pseudomonas aeruginosa*, as a model of lung infection in Cystic Fibrosis (CF), those fed with 3-cycle pressurized WPI had a significantly lower weight loss and mortality relative to mice fed either native WPI or casein [58]. Similar to the findings of another study [56], uninfected mice fed pWPI demonstrated no improvement in tissue GSH content as compared to native WPI feeding; however, *P. aeruginosa-infected* mice fed pWPI showed higher pulmonary concentrations of GSH when compared to infected mice fed either native WPI or casein fed mice. In a follow-up study, female C57BL/6 mice were fed either native whey or pressurized whey as a protein source for 4-week, followed by exposure to *P. aeruginosa* [59]. Lung bacterial burden was decreased in the mice receiving the pressurized whey, while neutrophilic oxidative burst and bacterial killing ability were enhanced.

In another animal study, piglets with DSS-induced colitis were randomized to receive to receive for 12 days complete isoenergetic liquid diets by gastrostomy, with three groups supplying 50% of protein requirement: pWPI, native WPI (nWPI), and skim milk (SM) plus a well-nourished SM group providing 100% of protein requirement. Protein fractional synthesis rate in the descending colon was not different among groups; however, histopathological scores were lowest in both WPI groups. PW-fed pigs had the lowest myeloperoxidase activity reflecting low neutrophil infiltration, and the highest total antioxidant activity as assessed via the ferric reducing ability of plasma assay. Proinflammatory cytokines (TNF-α, IL-8, and IL-18) in descending colon were most elevated in SM piglets, and lowest in the pWPI group. This pattern was repeated at the clinical level, as PW-fed pigs had less severe diarrhea compared with other protein restricted groups. In conclusion, pWPI-fed piglets showed antioxidant, and anti-inflammatory effects and decreased disease severity in this piglet model of pediatric IBD [60].

Healthy subjects provided with short-term intake of pWPI have shown rapid increases in lymphocyte GSH status within a 2-week period. The subjects receiving 45 g of pWPI supplements in addition to their normal diet showed increased lymphocyte GSH by 24% [61]. The increase in lymphocyte GSH concentrations with pWPI was similar to what was previously observed over a longer 3-mo period of 20 g of nWPI supplementation. The findings indicated that pWPI caused a six-fold faster increase in tissue GSH with one third total amount of protein supplementation [32]. The GSH-raising effects in the pWPI study are comparable with a similar open-label trial that tested an oral high dose of the GSH precursor prodrug *N*-acetyl-L-cysteine in 18 CF patients. This latter trial showed a 23% increase in GSH levels of blood neutrophils following a 4-wk supplementation period [62].

An open label clinical trial investigated the impact of 1-mo of supplementation with 30 g/d of pWPI in 27 CF adults and children [63]. Anthropometric measures, pulmonary function, serum C-reactive protein (CRP), and whole blood GSH tests were performed. The results of this study found that short-term supplementation of subjects with CF with pWPI had improved nutritional status (BMI z-score in children and BMI in adults) and lung function (% predicted FEV1 in children) although this study was limited by the short duration of supplementation, the small number of participants, and no control group [63]. In addition, the majority of patients with an initially elevated CRP showed a significant decrease when pWPI was supplemented, although whole blood GSH levels were unchanged. Thus, oral supplementation with pWPI in CF patients improved nutritional status and lung function and may also have additional beneficial anti-inflammatory effects. This study contrasts with previous work in CF subjects showing no change in nutritional status or lung function with 20 g nWPI supplementation over a 3-month supplementation period despite an enhancement of lymphocyte GSH levels [13].

A randomized, double-blind placebo-controlled study investigated the effects of pWPI supplementation (20 g/d) alone or in combination with an exercise training program in patients with chronic obstructive pulmonary disease (COPD) [64]. pWPI supplementation resulted in a significant increase in cycling endurance test time, as well as significant improvements in self-reported levels of fatigue while supplementation with the casein placebo did not [64]. In another human trial, colorectal surgical cancer patients consumed an oral nutrition drink before surgery, with either glucose alone or pWPI plus glucose. Leucine balance increased in postoperative patients receiving the pressurized whey protein plus glucose drink. Although leucine oxidation doubled, its appearance from protein breakdown decreased and as such was the major determinant of positive balance. The authors concluded that the oral drink based on pWPI and glucose reduced whole body protein breakdown so a positive protein balance occurred [65].

**Table 2:** Effects of pWPI in cell cultures, animal and human trials.

Study Objective	Study Design	Results	Reference
Effect of high hydrostatic pressure to improve *in vitro* digestibility of whey protein isolate (WPI) and exert glutathione (GSH)-enhancing and anti-inflammatory properties in CF cells.	Whey protein hydrolysates were generated using protocol mimicking human gastro-intestinal digestion. Cystic fibrosis (CF) cells were stimulated with tumor necrosis factor-alpha (TNF-α).	Improved *in vitro* digestibility; exerted GSH-enhancement by the release of novel peptides by gastrointestinal digestive enzymes; showed a trend to decrease interleukin-8 (IL-8) secretion in stimulated CF cells.	[47]
Effect of peptides released from the digestion of pressurized whey protein isolate (pWPI) to attenuate the inflammatory responses of CF cells.	Hydrolysates of pWPI were generated *in vitro* and tested for anti-inflammatory properties via the suppression of IL-8 production in CF and non-CF cells.	Suppressed IL-8 production stimulated by lipopolysaccharide (LPS); reduced LPS binding to surface Toll-like receptor (TLR)4, while pWPI tended to more potently increase extracellular antioxidant capacity.	[66]
Effect pWPI *vs.* native whey protein isolate (nWPI) hydrolysates in Caco-2 cells exposed to hydrogen peroxide (H_2_O_2_).	Cells were cultured with varying concentrations of pWPI or nWPI hydrolysates in the presence of H_2_O_2_. Outcome measures included IL-8, intracellular reactive oxygen species (ROS) and the ferric reducing ability of plasma (FRAP) assay.	IL-8 secretion and ROS generation was diminished in concert with enhanced FRAP activity; H_2_O_2_-induced IL-8 showed greater inhibition with 2000 µg·mL^−1^ of pWPI (50%) *vs.* nWPI (30%); H_2_O_2_-induced ROS formation was reduced by 76% for pWPI, as compared to 32.5% with nWPI hydrolysates.	[67]
Effect of pWPI to enhance the host ability to clear *P. aeruginosa* infection compared with nWPI.	Female C57BL/6 mice were fed 20% of either nWPI or pWPI as a protein source for 4-week, followed by exposure to *P. aeruginosa*.	Inhibited airway protein oxidation; increased the ability of destroying bacterial leukocytes and oxidative burst in response to phagocytized bacteria in the pressurized nWPI-fed mice.	[59]
Effect of pWPI, nWPI, and skin milk (SM) in piglets with DSS-induced colitis.	Female piglets received 50% of protein requirement as pWPI, nWPI, and SM and a control diet (100% SM). Protein synthesis and proinflammatory cytokines (TNF-α, IL-8, and IL-18) was studied on day 12 with a 6-h infusion of tracer L-[ring-^2^H_5_]phenylalanine.	Decreased histopathological scores in both WPI groups; lowest myeloperoxidase activity and the highest total antioxidant activity in pWPI-fed piglets; elevated TNF-α, IL-8, and IL-18 in descending colon in the SM piglets and lowest in the pWPI group.	[60]
Effect of pWPI on healthy subjects in lymphocyte GSH status.	During a 2-week period, 31 subjects received 45 g of pWPI supplements in addition to their normal diet.	Increased lymphocyte GSH by 24%.	[61]
Effect of pWPI supplementation in children and adults with CF.	During 1-month, 27 patients with CF (9 children, 18 adults) consumed different doses of pressurized whey: 20 g/day in patients less than 18 years of age and 40 g/day in older patients.	Improved in lung function (forced expiratory volume in 1 s); elevated C-reactive protein (CRP) showed a reduction; decreased phytohemagglutinin-stimulated IL-8 responses in the adults.	[63]
Effect of pWPI alone or in combination with an exercise training program in patients with chronic obstructive pulmonary disease (COPD).	For a 16-week period, 22 patients consumed 20 g/day pWPI or casein. Parameters used to evaluate the effects of treatments: Chronic Respiratory Questionnaire (CRQ) and constant work rate cycle endurance test (CET).	Increased CET time and improved fatigue and emotional control scales of the CRQ in the pWPI-only; improved in the dyspnea and the mastery scales of the CRQ in both groups.	[64]
Effect of glucose alone or containing pWPI on colorectal surgical cancer patients consumed an oral nutrition drink before surgery.	A constant infusion of D-[6,6-^2^H_2_]glucose and L-[1-^13^C]leucine were conducted to determine glucose rate of appearance and whole body protein turnover in 17 patients at the fasted and fed states.	Increased leucine balance in postoperative patients receiving the pWPI plus glucose drink; reduced whole body protein breakdown so a positive protein balance occurred.	[65]

Taken together, the unfolding of the globular structure of whey proteins and their increased proteolysis following pressurization has been related to enhanced anti-inflammatory, antioxidant and anabolic effects as the major outcomes of the human and animal feeding trials involving pWPI. Contradictory findings, however, have been seen in terms of tissue GSH and lipid peroxidation outcomes, which could be related to the animal model tested. There remain unanswered questions and controversy regarding how variations in the manufacture and isolation of WPI and the resultant varying compositional protein profiles can affect the bioactivities of pWPI.

## 5. Conclusions

Hyperbaric pressure treatment of whey protein results in protein unfolding. This enhances whey digestibility and results in a change in the spectrum of peptides produced. Supplementation with pWPI enhances growth, protein accretion, and antioxidant protection, while reducing inflammation and enhancing the response to infection. The results to date point to potential application for enhancing growth without antibiotics for animals, and a variety of clinical applications, ranging from pre- and post-operative nutritional recovery, enhancing muscle accretion and function, to reducing inflammation.
